# Supplementary material for: Real-time 3D single molecule tracking
Source: Nat Commun. 2020 Jul 17;11:3607. doi: 10.1038/s41467-020-17444-6 (PMC7368020; doi:10.1038/s41467-020-17444-6)
Supplement: Supplementary file 3 — Description of Additional Supplementary Files [file 41467_2020_17444_MOESM3_ESM.pdf]

Description of Additional Supplementary Files

File Name: Supplementary Data 1
Description: Measured step response data of the piezoelectric nanopositioner.

File Name: Supplementary Movie 1
Description: 3D-SMART tracking of a single Atto 647N molecule in 90 wt% glycerol. The top panel shows the molecule's real-time trajectory and the bottom left panel shows the fluorescence intensity as a function of time. The real-time photon readout at the individual XY laser scan positions is displayed in the bottom right.

File Name: Supplementary Movie 2
Description: Real-time intensity traces with feedback (top) off and (bottom) on. The real-time photon readouts at the individual XY laser scan positions are displayed on the right.

File Name: Supplementary Movie 3
Description: BSA-Atto 647N photo-blinking captured with 3D-SMART tracking. The top panel shows the molecule's real-time trajectory and the bottom left panel shows the fluorescence intensity as a function of time. The real-time photon readout at the individual XY laser scan positions is displayed in the bottom right.

File Name: Supplementary Movie 4
Description: Real-time tracking of 1385 bp dsDNA tracking in PBS. The top panel shows the molecule's real-time trajectory and the bottom left panel shows the fluorescence intensity as a function of time. The real-time photon readout at the individual XY laser scan positions is displayed in the bottom right.

File Name: Supplementary Movie 5
Description: DFHBI-1T labeled mRNA tracking in 75 wt% glycerol. The top panel shows the molecule's real-time trajectory and the bottom left panel shows the fluorescence intensity as a function of time. The real-time photon readout at the individual XY laser scan positions is displayed in the bottom right.

File Name: Supplementary Movie 6
Description: Real-time measurement of transcription on a single, freely diffusing dsDNA. The top panel shows the dsDNA molecule's real-time 3D trajectory. The trajectory is labelled with a green sphere when mRNA (fastFISH) signal is observed. The bottom panels show the intensity of Atto 647N labeled dsDNA (red) and the readout of the ssDNA (green). The real-time photon readout at the individual XY laser scan positions is displayed in the bottom right.
